# Antioxidant Strategies for Inherited Retinal Degenerations in the Era of Gene Therapy

**DOI:** 10.3390/antiox15101229

**Published:** 2026-09-23

**Authors:** Yiliu Yang, Qin Chen, Jingxin Zeng, Licong Liang, Zhiyan Tao, Kaiqin She, Chengda Ren, Fang Lu

**Affiliations:** Department of Ophthalmology, West China Hospital, Sichuan University, Chengdu 610041, China; y1161606786@163.com (Y.Y.); chenqin@wchscu.cn (Q.C.);

**Keywords:** inherited retinal degenerations, oxidative stress, antioxidant therapy, gene therapy

## Abstract

Inherited retinal degenerations (IRDs) are a genetically heterogeneous group of disorders characterized by progressive loss of photoreceptors and retinal pigment epithelium, leading to irreversible vision impairment. Although gene therapy has transformed the therapeutic outlook for IRDs, its efficacy remains limited by gene and variant specificity, a narrow treatment window dependent on viable target cells, and the persistence of retinal degeneration even after restoration of gene function. In this review, we summarize current gene-based therapeutic strategies, critically examine oxidative stress as a shared but heterogeneous downstream mechanism across IRDs, and review antioxidant and redox-modulating interventions with particular emphasis on the distinction between preclinical and clinical evidence. This review highlights that oxidative stress contributes to inflammation, metabolic dysfunction, and cellular damage during disease progression and may be exacerbated by interventions associated with gene therapy—including vector-induced immune activation, transgene-related cellular stress, and delivery-associated tissue injury. However, direct evidence supporting antioxidant–gene therapy combinations remains predominantly preclinical, and their clinical efficacy has not yet been established. Disease- and stage-specific integration of antioxidants and redox modulation may help preserve viable retinal cells, support retinal homeostasis during genetic rescue, and potentially improve treatment durability.

## 1. Introduction

Inherited retinal degenerations (IRDs) represent a group of clinically and genetically heterogeneous disorders and are a major cause of irreversible vision loss and blindness worldwide [1]. Although caused by diverse pathogenic variants and presenting with distinct clinical phenotypes, IRDs including retinitis pigmentosa (RP), Leber congenital amaurosis (LCA), and Stargardt disease ultimately lead to progressive loss of photoreceptors and retinal pigment epithelium (RPE), retinal thinning, and irreversible visual impairment [2,3,4], therefore imposing a significant burden on patients, families, and healthcare systems [5].

The emergence of gene therapy has transformed the treatment landscape for IRDs. Numerous gene-based therapies for IRDs have entered clinical trials, one of which has already received regulatory approval [6,7]. Despite significant advances in gene therapy, challenges such as vector-related toxicity, immune responses, and limited therapeutic windows remain unresolved [6,8]. Moreover, current gene therapies target only a very narrow range of genes and variant types, leaving many patients with IRDs caused by other genetic defects lacking suitable treatment options. More importantly, correction of the gene defect does not necessarily restore a healthy retinal environment. Rescued photoreceptors may still be exposed to microenvironmental abnormalities including oxidative stress, mitochondrial dysfunction, inflammation, and metabolic imbalance, allowing retinal degeneration to continue [9,10,11]. Therefore, in the era of gene therapy, there is growing recognition of the need for complementary therapeutic strategies to preserve retinal function, slow disease progression, alleviate additional burdens associated with gene therapy, and extend its efficacy.

Oxidative stress is a common downstream pathological process across multiple IRDs. Due to the high oxygen consumption, prolonged exposure to light, and physiological characteristics such as abundant mitochondria and polyunsaturated fatty acids, the retina is particularly vulnerable to oxidative damage [12]. The RPE is similarly exposed to a persistent oxidative burden because of its continuous phagocytosis of photoreceptor outer segments and long-term accumulation of lipofuscin and other photo-oxidative products [13].

Antioxidant therapies have been shown to delay retinal degeneration in various preclinical models, while N-acetylcysteine (NAC) treatment in RP patients has further demonstrated promising safety and potential benefits in early clinical studies [14,15,16,17]. The ability to delay disease progression gives antioxidants the potential to provide more living cells for gene therapy and extend the therapeutic window. However, antioxidant therapy alone may be limited by dosing requirements, the need for prolonged treatment, uncertain effects on retinal structure and visual function, and an inability to address the underlying genetics. Therefore, combining antioxidant and gene therapies may yield greater benefits than either approach alone.

In this review, we summarize the current progress, limitations and retinal adverse effects of gene therapy for IRDs, examine the role of oxidative stress in the pathogenesis of different IRDs, discuss current antioxidant strategies and their therapeutic potential, and highlight the timing, challenges, and prospects of antioxidant–gene therapy combinations for IRDs. We propose that future treatments for IRDs may require a combinatorial framework in which genetic correction by gene therapy and microenvironmental stabilization by antioxidants work together to achieve more durable and broadly applicable visual outcomes. The overall conceptual framework of this review, including the relationship between gene therapy, oxidative stress, treatment-associated retinal stress, and the proposed timing of antioxidant intervention, is summarized in Figure 1.

## 2. Gene Therapy for IRDs: Achievements and Unmet Therapeutic Challenges

### 2.1. Current Gene Therapy Strategies

IRDs represent one of the most attractive targets for gene therapy because of their predominantly monogenic nature, well-characterized genetic mechanisms, and the relative accessibility and immune privilege of the eye [5]. Over the past two decades, multiple gene-based strategies for IRDs have been developed, including gene augmentation, genome editing, RNA-targeted therapies, modifier-gene approaches, and optogenetic strategies (Table 1) [18,19].

The optimal gene-based strategy depends not only on the causative gene but also on the underlying pathogenic mechanism of the disease-causing variant. Most autosomal recessive and X-linked IRDs are caused by loss-of-function variants and are therefore suitable for gene augmentation [1,39]. Some autosomal dominant disorders caused by haploinsufficiency, such as *PRPF31*-associated RP, may also benefit from gene augmentation [23,40]. In contrast, dominant-negative or gain-of-function variants, such as *RHO*-associated autosomal dominant RP, generally require suppression or correction of the pathogenic allele through genome editing or RNA interference [41,42]. In addition, modifier-gene and optogenetic therapies are largely independent of the specific causative variant and inheritance mode: the former modulates shared downstream disease pathways [36], whereas the latter restores light responsiveness in surviving retinal neurons regardless of photoreceptor function [38].

Among these approaches, gene augmentation using adeno-associated virus (AAV) vectors remains the most clinically advanced strategy [43]. By delivering a functional copy of a defective gene into target retinal cells, AAV-based supplementation aims to restore protein expression and recover cellular function without intentional genomic integration [44]. Voretigene neparvovec (Luxturna), an AAV2-based gene augmentation therapy, has been approved for biallelic *RPE65*-associated retinal dystrophy by the U.S. Food and Drug Administration (FDA) [20,44]. Beyond approved products, dozens of AAV-based gene therapies targeting genes such as *RPGR, GUCY2D, CNGA3, CNGB3*, and *RS1* are undergoing clinical evaluation, the majority of which are in early-stage (phase 1/2) trials [8,21,45,46,47,48,49].

### 2.2. Remaining Limitations and Challenges of AAV-Mediated Gene Therapy

#### 2.2.1. Limitations in Therapeutic Efficacy and Broad Applicability

##### Need for Individualized Therapeutic Design

Despite remarkable progress, several challenges continue to limit the broader application and complete functional recovery of AAV-mediated gene therapy for IRDs. One major obstacle is the extreme genetic heterogeneity of IRDs. Although gene augmentation therapy can be applied to different loss-of-function variants within the same gene, genome editing and RNA editing strategies are often variant-specific and therefore highly individualized [6,7].

##### Vector Packaging Capacity Limitations

Large genes that exceed the packaging capacity of a single AAV vector require dual- or multi-AAV delivery approaches. These approaches depend on efficient co-transduction of the same target cell and correct reconstitution of the split transgene, which generally results in lower productive expression than that achieved with a single vector [50,51,52]. Incorrect or incomplete reconstitution may also generate nonproductive or unintended RNA or protein products, depending on the specific vector design and reconstitution mechanism [52,53]. In addition, achieving sufficient co-transduction with multiple vector components may require greater overall vector exposure [51,54], potentially increasing dose-related AAV immune and inflammatory responses [55].

##### Dependence on Viable Target Cells

Another important limitation shared by many gene-based therapeutic approaches is their dependence on the presence of viable target cells, especially for gene augmentation, genome editing, and RNA-targeted correction [1,2]. As preservation of viable retinal target cells is essential for functional rescue, the therapeutic efficacy of these approaches is closely associated with disease stage and remaining retinal structure [1,56]. For instance, both preclinical studies in *RPE65*-associated LCA animal models and subsequent clinical trials have demonstrated greater therapeutic benefit in eyes with preserved photoreceptors [56].

##### Limited Durability and Efficacy

The clinical benefits of gene correction may be less durable than initially anticipated, with early improvements not necessarily translating into sustained functional gains. A meta-analysis of clinical studies in patients with *RPE65*-associated LCA found a significant improvement in best-corrected visual acuity (BCVA) at 1 year after gene therapy; however, this benefit appeared to diminish over time, with essentially no significant difference in BCVA between treated and untreated eyes after 2–3 years of follow-up [9].

Moreover, the efficacy of gene therapy is closely related to the property and location of targeted protein. The *RPE65* gene therapy and the recently reported scAAV8-*hRS1* therapy for X-linked retinoschisis represent two relatively favorable examples: the diffusible retinoid substrates and products of RPE65 allow transduced RPE cells to support neighboring photoreceptors, whereas *RS1* encodes a secreted extracellular protein that can act beyond the transduced cell [9,20,21]. In many other IRDs, however, the causative defect is cell autonomous and involves intracellular or membrane-associated proteins in RPE cells or photoreceptors [1,2]. Therefore, for most IRDs, better therapeutic efficacy requires higher transduction efficiency and a broader transduction range, which are strongly dependent on the delivery route, vector platform and target cell type. In nonhuman primates, subretinal AAV8 similarly provides substantially greater photoreceptor and RPE transduction than intravitreal delivery [57], while the coverage of subretinal injection has been shown to be limited (only 10–20% of the total retinal area) in early rodent studies [58].

Overall, the application and effectiveness of gene therapy can be limited by genetic heterogeneity, disease stage, the viability of target cells and durability. Therefore, complementary strategies that may help preserve residual retinal cells and potentially prolong the functional benefits of gene-based therapies deserve further investigation.

#### 2.2.2. Therapy-Associated Toxicity and Cellular Stress

Progressive structural changes have been reported after subretinal AAV gene therapy, including localized retinal thinning, RPE alterations, and chorioretinal atrophy [9,10,20,59,60,61]. Their mechanisms remain incompletely understood and potentially involve interactions among vector-related immune and inflammatory responses, transgene-induced cellular stress, and delivery-associated mechanical injury [20,59]. These observations raise the possibility that therapeutic interventions may impose additional stress on an already vulnerable retinal environment.

##### AAV-Induced Immune and Inflammatory Stress

The eye has traditionally been regarded as an immune-privileged organ. However, progressive photoreceptor degeneration in IRDs disrupts retinal architecture, impairs the blood–retinal barrier (BRB), and promotes local immune activation, thereby increasing retinal susceptibility to inflammation and further degeneration [62]. Although AAV vectors are generally considered relatively safe, they can still trigger innate and adaptive immune responses depending on the vector dose, serotype, and route of administration [8,63,64].

AAV administration may engage components of retinal innate immunity, including the complement system and microglia. Recent preclinical studies of intravitreal AAV delivery demonstrated activation of complement-related pathways and accumulation of C3a in ocular fluids, accompanied by microglial activation and peripheral leukocyte infiltration [65]. Microglial responses have also been observed after subretinal AAV administration in large-animal models, particularly at higher vector doses [66,67]. As resident immune cells of the retina, microglia can internalize AAV-derived material and may contribute to antigen presentation and recruitment of peripheral immune cells [68].

The AAV capsid and the gene-expression cassette both contribute to host immune activation. AAV capsid recognition can trigger humoral and cellular immune responses and promote the release of pro-inflammatory mediators [63]. In addition, unmethylated CpG motifs within inverted terminal repeats (ITRs) and transgene cassettes can activate TLR9–MyD88 signaling and type I interferon responses, further amplifying inflammatory signaling [63,69,70]. These inflammatory responses may further interact with oxidative stress because activated microglia can generate reactive oxygen species (ROS) and reactive nitrogen species [71]. However, a direct link between AAV-induced inflammation and oxidative stress has not been conclusively demonstrated.

##### Transgene-Induced Cellular Stress

In addition to vector-related effects, transgene expression itself can impose cellular stress. Photoreceptors are highly dependent on tightly regulated protein synthesis and trafficking for outer-segment maintenance [72]. AAV-mediated overexpression of wild-type rhodopsin impairs retinal function in normal mice, indicating a limited tolerance for excessive expression even of a native photoreceptor protein [73]. One potential mechanism involves endoplasmic reticulum (ER) stress. High-level AAV-mediated HMOX1 expression induces DDIT3-associated ER stress and photoreceptor degeneration [74]. Separately, ER stress is well established to aggravate oxidative stress through mechanisms including glutathione (GSH) depletion and increased ROS production [75]. However, the extent to which this ER–redox coupling contributes to transgene-associated retinal toxicity remains unclear.

An emerging strategy to mitigate expression-related cellular stress is the use of regulatable AAV expression systems. Tetracycline-responsive AAV vectors have enabled reversible and repeated control of transgene expression in the retina, including long-term regulation in nonhuman primates [76,77,78,79]. In *RPE65*-deficient dogs, doxycycline-regulated AAV-*RPE65* allowed induction and de-induction of retinal function, although the achieved expression level was insufficient to restore vision [80]. Such systems could allow transgene expression to be titrated within a therapeutic range and reduced if expression-related toxicity emerges. However, additional vector cargo requirements and potential immunogenicity of regulatory proteins remain important barriers [81].

##### Subretinal Delivery-Associated Mechanical Stress

For AAV therapies targeting photoreceptors and RPE cells, subretinal delivery is commonly used because it places the vector directly adjacent to target cells and can achieve efficient transduction at relatively low doses, while potentially limiting systemic and humoral immune exposure [57]. However, this approach requires transient separation of the neurosensory retina from the RPE and is therefore intrinsically invasive. Previous studies have suggested that transient retinal detachment or edema caused by subretinal injection can impose additional stress on retinal tissue, potentially associated with later retinal thinning [9].

In addition, excessive injection pressure or prolonged stay in the subretinal space may disrupt the integrity of the retina and lead to complications such as retinal overstretching, focal thinning, macular hole formation, or RPE injury [82,83,84,85,86]. Direct evidence linking injection-induced mechanical stress to oxidative injury in retinal gene therapy remains limited. Nevertheless, retinal detachment and the transient separation of the neurosensory retina from the RPE have been associated with increased ROS generation, protein oxidation, and NADPH oxidase (NOX) activity [87,88,89]. Thus, transient subretinal delivery may impose additional redox stress, although not yet clinically established.

## 3. Oxidative Stress as a Shared Downstream Pathway in IRDs

Although IRDs arise from highly diverse genetic defects, many of them share common secondary pathways that continue to drive retinal degeneration beyond the initial genetic defect. Among these, oxidative stress has emerged as one of the most important and broadly shared mechanisms [12].

Oxidative stress refers to an imbalance between the production and clearance of ROS [12]. ROS are highly reactive oxygen-containing molecules generated as byproducts of aerobic metabolism. Major sources of ROS include electron leakage from the mitochondrial respiratory chain and enzymatic reactions catalyzed by NOXs, both of which generate superoxide anions (O_2_^•−^) [13]. Superoxide is rapidly converted into hydrogen peroxide (H_2_O_2_), which can diffuse across cellular and mitochondrial membranes [90]. In the presence of transition metals such as iron, H_2_O_2_ undergoes Fenton reactions to produce highly reactive hydroxyl radicals (HO^•^) [91]. Nitric oxide (NO^•^), generated by various enzymatic pathways, can react with ROS to form reactive nitrogen species such as peroxynitrite (ONOO^−^), further amplifying oxidative damage [92]. In addition, ER, microsomes and peroxidases can also generate O_2_^•−^ and H_2_O_2_ [93]. ROS play an important role in intracellular signaling, immune regulation, and cellular homeostasis. However, excessive ROS accumulation or impaired antioxidant defenses can damage proteins, lipids and nucleic acids, which in turn lead to structural and functional cell impairment [94].

### 3.1. Anatomical and Physiological Basis for Retinal Vulnerability to Oxidative Stress

The retina is susceptible to oxidative stress because several anatomical and metabolic features collectively create a high and sustained oxidative burden. Photoreceptors and RPE have high energetic demands associated with phototransduction, ion homeostasis, and continuous outer-segment renewal [95,96]. At the same time, the retina is continuously exposed to visible light required for vision. Light can interact with endogenous photosensitive molecules, including retinaldehyde-derived compounds and RPE lipofuscin bisretinoids such as A2E, promoting photo-oxidative reactions and additional ROS generation [97].

Consequently, photoreceptors and RPE cells contain abundant mitochondria with high metabolic activity, which also contributes to mitochondrial ROS generation [98]. Under pathological conditions, impaired electron transport in mitochondria can simultaneously reduce ATP production and increase ROS generation [99]. Using the mitochondria-targeted probe MitoB, increased mitochondrial H_2_O_2_ was detected before overt photoreceptor loss in several genetically distinct RP models, including rd1, rds, Rpgr^−/−^, and Cln6^nclf^ mice [100], indicating that mitochondrial ROS generation may be one of the earliest pathological features of IRDs [101].

In addition, photoreceptor outer-segment membranes are enriched in polyunsaturated fatty acids, particularly docosahexaenoic acid (DHA) [102]. Although DHA is essential for membrane organization and photoreceptor function, its high degree of unsaturation makes these membranes particularly susceptible to ROS-induced lipid peroxidation [102]. Lipid peroxidation can compromise membrane integrity and generate reactive lipid products that further propagate oxidative injury [13,103].

RPE cells are also highly susceptible to oxidative stress. Mature RPE cells are long-lived cells with limited regenerative capacity, and they continuously phagocytose photoreceptor outer segments while supporting the visual cycle and photoreceptor metabolism [104]. Incomplete degradation of outer-segment-derived material contributes to the age-dependent accumulation of lipofuscin, whose bisretinoid components can undergo photo-oxidation and further compromise lysosomal and cellular homeostasis [105]. The retina also contains redox-active iron, which can catalyze hydroxyl-radical formation and promote lipid peroxidation, providing an additional source of oxidative injury [106].

### 3.2. Oxidative Stress as an Amplifier of Inflammation, Metabolic Failure, and Cell Death

Oxidative stress in IRDs does not act in isolation. In the degenerating retina, redox imbalance is closely linked to multiple pathogenic pathways [12,13]. Oxidative injury can promote inflammatory cascades by activating microglia, Müller glia, and infiltrating immune cells, which can subsequently generate additional reactive oxygen and nitrogen species [107,108]. At the signaling level, excessive ROS can activate redox-sensitive NF-κB pathways and promote inflammasome signaling, including NLRP3 activation, thereby enhancing the production of pro-inflammatory mediators [109,110]. Inflammatory signaling and glial activation can, in turn, increase mitochondrial- and NOX-derived ROS production, providing a mechanistic basis for bidirectional coupling between oxidative stress and inflammation [71,111].

In *rd10* mice, oxidative DNA damage in microglia activates MUTYH-dependent base-excision repair and PARP signaling, promoting microglial activation, TNF-α production, and subsequent photoreceptor degeneration [112]. Consistently, treatment with the antioxidant N-acetylcysteine (NAC) suppresses both oxidative damage and the expression of inflammatory cytokines and chemokines, reduces microgliosis, and attenuates photoreceptor death [113]. In addition, activated microglia and reactive Müller glia release TNF-α, IL-1β, IL-6, chemokines, and other cytotoxic mediators that can further disturb mitochondrial function, increase oxidative/nitrosative stress, promote aberrant phagocytosis, and amplify photoreceptor injury [12]. Recent studies in *Rho^P23H/+^* and *rd10* models further demonstrate that attenuation of glial activation and inflammatory signaling is associated with restoration of antioxidant defense pathways, while simultaneous targeting of neuroinflammation and oxidative stress provides greater photoreceptor protection than either intervention alone [109,114]. Together, these findings support a feed-forward loop in which oxidative stress and inflammation mutually reinforce retinal degeneration.

Beyond inflammation, oxidative stress is also closely linked to metabolic dysfunction [115]. Photoreceptors depend heavily on aerobic glycolysis and mitochondrial oxidative phosphorylation, while glucose metabolism through the pentose phosphate pathway provides NADPH required to maintain GSH- and thioredoxin-dependent antioxidant systems [116,117,118,119]. Disturbance of mitochondrial function or glucose utilization therefore affects both ATP production and cellular reducing capacity. As antioxidant reserves decline, photoreceptors become increasingly vulnerable to further oxidative injury. Studies of the rod-derived cone viability factor (RdCVF), its longer isoform RdCVFL and thioredoxin-interacting protein (TXNIP) pathways provide particularly clear examples of this metabolic–redox coupling, showing that glucose utilization, mitochondrial homeostasis, and antioxidant defense are closely linked to photoreceptor survival [117,118,120,121].

When oxidative stress becomes sustained or excessive, it can also shift cells from reversible dysfunction toward cell death [12,75,105]. Elevated intracellular ROS and lipid peroxidation can promote mitochondrial dysfunction and apoptotic signaling [122], while depletion of GSH-dependent antioxidant defenses and uncontrolled lipid peroxidation can lower the threshold for ferroptotic cell death [13,16]. Oxidative and mitochondrial stress are also closely coupled to disruption of autophagic homeostasis [122]. Recent analyses across genetically distinct IRD mouse models identified mitochondrial stress and impaired autophagic clearance as early events preceding photoreceptor loss and subsequent necroptotic cell death [123]. Disruption of protein folding and redox homeostasis can activate the unfolded protein response, whereas prolonged ER stress can further increase ROS production and promote cell-death signaling [75,105,124]. In *RHO*-associated RP, perturbation of the IRE1α and PERK branches of the unfolded protein response alters photoreceptor proteostasis and autophagic processing and accelerates retinal degeneration [125]. Together, these interactions help explain why oxidative stress can accelerate retinal degeneration even when it is not the initiating cause of disease.

### 3.3. Oxidative Stress Across IRDs

Because of the genetic heterogeneity of IRDs, the mechanisms underlying disease initiation and progression vary, and oxidative stress may arise from different upstream disturbances. In typical RP, rod photoreceptors degenerate first, followed by secondary cone loss [1]. A well-supported mechanism for cone loss is that extensive rod loss reduces oxygen consumption in the outer retina, resulting in relative hyperoxia and subsequent oxidative injury to surviving cones [126]. Shen et al. demonstrated progressive oxidative modifications, including lipid peroxidation products (acrolein, 4-HNE) and protein/DNA damage, in cones of a transgenic RP pig following extensive rod loss [127]. Subsequently, NOX inhibition with apocynin reduced superoxide and oxidative damage, preserved cone density, and maintained photopic electroretinography (ERG) responses in *rd1* and *Rho-Q344X* models, implicating NOX-derived ROS in secondary cone loss [128]. More recently, lowering inspired oxygen in *rd10* mice decreased retinal superoxide and preserved cone structure and function, directly supporting hyperoxia as an upstream driver of oxidative stress after rod loss [129].

By contrast, oxidative stress in Stargardt disease is closely linked to abnormal retinoid handling and accumulation of photoreactive bisretinoids, such as A2E, in the RPE [130]. A2E can generate ROS upon short-wavelength light exposure [131]. In *Abca4*^−/−^ mice, increased expression of oxidative-stress-response genes encoding superoxide dismutase 1 (SOD1), catalase (CAT), heme oxygenase-1, and glutathione S-transferases, is accompanied by increased lipid-peroxidation products, supporting a close association between bisretinoid accumulation and chronic oxidative stress in the RPE [132]. Another inherited macular disorder, Best disease, represents a different RPE-centered mechanism. Although autofluorescent material also accumulates in Best disease, patient-derived *BEST1*-mutant RPE cells exhibit impaired photoreceptor-outer-segment degradation, altered Ca^2+^ homeostasis, and increased oxidative-stress responses after chronic outer-segment exposure [133].

Marked heterogeneity is also evident among different kinds of LCA. In *RPE65* deficiency, the primary molecular defect is impaired visual-cycle chromophore regeneration [1], while available evidence suggests that oxidative stress acts mainly as a secondary amplifier of photoreceptor degeneration [134]. Oxidative-stress responses are increased in *Rpe65*-deficient retinas and can be reduced by suppression of thyroid-hormone signaling or antioxidant treatment [134,135]. In contrast, in *Nmnat1^V9M/V9M^* mice, progressive accumulation of the oxidative DNA lesion 8-oxo-dG is accompanied by DNA double-strand breaks and apoptosis, showing a more direct relationship between redox damage and photoreceptor death [16].

Moreover, retinal antioxidant capacity is also influenced by aging. In aged RPE, the inducibility of Nrf2-dependent antioxidant responses to oxidative challenge is impaired, accompanied by greater oxidative damage, while aging is also associated with mitochondrial dysfunction and weakened antioxidant defenses [136]. These age-related changes may lower the ability of the retina to buffer disease-associated oxidative stress, particularly in slowly progressive IRDs and at later disease stages.

## 4. Antioxidant and Redox-Modulating Therapeutic Strategies for IRDs

Given the central role of oxidative stress in pathogenesis and progression of IRDs, multiple antioxidant and redox-modulating therapies have been applied and have shown promising results. These strategies are classified primarily according to their predominant therapeutic mechanism rather than solely by chemical origin or route of administration. Notably, the categories are not mutually exclusive because many antioxidant agents are pleiotropic. Compounds are assigned according to the mechanism emphasized in the present review. Broadly, these approaches include small-molecule and nutritional interventions, enhancement of endogenous antioxidant systems, metabolic–redox modulation, and gene-based strategies. The following sections summarize antioxidant and redox-modulating strategies investigated across preclinical and clinical studies, whereas Table 2 specifically highlights those that have progressed to human evaluation.

Notably, antioxidant interventions that have reached clinical evaluation in IRDs are dominated by orally administered small-molecule or nutritional approaches. Strategies based on direct activation of endogenous antioxidant pathways or gene-based redox modulation remain predominantly preclinical.

### 4.1. Small-Molecule and Nutritional Approaches

#### 4.1.1. Thiol Donors and GSH Precursors

GSH is a major endogenous antioxidant that maintains cellular redox homeostasis [13,147], and its synthesis is strongly influenced by cysteine availability [148]. NAC, a cysteine derivative with thiol groups, primarily supports intracellular antioxidant capacity by providing cysteine for GSH synthesis [12]. Its free thiol can also react directly with selected oxidant species [12]. Lee et al. first demonstrated the protective effects of oral NAC on cone density and function in *rd10* and *rd1* mice, and the significantly higher cone density in the 7 mg/mL NAC group lasted for up to 6 months in *rd10* mice [149]. Based on these findings, a phase 1 clinical trial of NAC (FIGHT RP, NCT03063021) was subsequently conducted. The results showed varying degrees of improvement in BCVA across all three dose groups, with a statistically significant improvement in mean macular sensitivity observed in the 1800 mg group [17,137]. However, no significant changes were observed in ellipsoid zone (EZ) width or fundus autofluorescence [137]. The ongoing NAC Attack trial (NCT05537220) is further assessing whether long-term oral NAC can slow retinal degeneration in RP. However, orally administered NAC undergoes deacetylation in the gastrointestinal tract and hepatic first-pass metabolism [150], resulting in a relatively low bioavailability of approximately 4–9.1% [151]. This may also explain the relatively high oral dose used in the FIGHT RP trial, with some participants experiencing dose-related gastrointestinal adverse reactions [137].

To overcome the limited bioavailability of NAC, several other cysteine derivatives, including N-acetylcysteine amide (NACA) and N-acetyl-L-cysteine ethyl ester (NACET), have been developed. NACA is the amide form of NAC and exhibits greater lipophilicity and membrane permeability than NAC [152]. Schimel et al. and Carver et al. demonstrated the antioxidant and protective effects of NACA in mouse models of light-induced retinal damage and in human RPE cells exposed to H_2_O_2_, respectively [153,154]. Wood et al. further showed that NACA has stronger antioxidant activity than NAC in cultured mouse retinal cells [155]. These preclinical findings supported the development of NPI-001, an oral NACA formulation, for RP associated with Usher syndrome. A randomized placebo-controlled Phase I/II study (SLO-RP, NCT04355689) enrolled 49 patients and completed 24-month follow-up. Sponsor-reported results indicated that NPI-001 slowed photoreceptor loss by more than 50% over two years, as assessed by EZ area, with generally favorable tolerability [156]. However, peer-reviewed efficacy results have not yet been published.

NACET represents another cysteine derivative with relatively high bioavailability [157]. NACET shows greater cellular permeability than NAC and increases intracellular cysteine and GSH levels in oxidatively stressed RPE cells [158]. Realini et al. demonstrated that NACET strongly stimulates the expression and activity of nuclear factor erythroid 2-related factor 2 (Nrf2) in RPE cells, an essential regulator of oxidative stress response [159]. More recently, Appell et al. reported that topical delivery of NACET using a gel-forming eye drop achieved photoreceptor protection and increased photopic b-wave amplitude in a chemically induced rat model of RP and in *rd10* mice [160]. However, further work is still needed to address issues including the stability and storage of NACET formulations [160].

#### 4.1.2. Micronutrients and Nutritional Lipids

Micronutrients and nutritional lipids have long been investigated as accessible approaches to modulate retinal oxidative stress. Classical antioxidant micronutrients include vitamins C and E and the xanthophyll carotenoids lutein and zeaxanthin. In *rd1* mice, combined administration of α-tocopherol, ascorbic acid (vitamin C), α-lipoic acid, and Mn (III) tetrakis (4-benzoic acid) porphyrin reduced oxidative damage and increased cone density and function, providing early proof that nutritional redox modulation can protect photoreceptors [161]. Vitamin E alone also delayed retinal degeneration in photoreceptor degeneration rat models, consistent with its ability to interrupt lipid-peroxidation chain reactions [162]. However, the efficacy of vitamin C as an isolated intervention has not been established in clinical IRD studies, while the findings of therapeutic vitamin E in patients have been disappointing. In a randomized 2 × 2 factorial trial involving 601 patients with RP, supplementation with 400 IU/day vitamin E was associated with a faster decline in cone electroretinographic amplitude rather than a protective effect [142].

Lutein and zeaxanthin form the macular pigment and may protect the retina by filtering short-wavelength light, quenching singlet oxygen, and limiting photo-oxidative damage [163,164]. In *rd10* and *Abca4*^−/−^*/Bco2*^−/−^ mice, supplementation with lutein and/or zeaxanthin delayed photoreceptor degeneration, reduced A2E-related bisretinoid accumulation, and improved retinal or visual function [165,166]. However, clinical studies in RP, Usher syndrome, Stargardt disease, and cone–rod dystrophy demonstrated increased serum lutein and, in some patients, macular pigment without consistent improvements in visual function [144,146]. A subsequent 4-year randomized trial in RP found no significant benefit in the primary visual-field endpoint, although a secondary endpoint suggested a modest slowing of visual-field decline with lutein, indicating that its potential disease-modifying effects remain unproven [138].

DHA is one of the most abundant polyunsaturated fatty acids in photoreceptor outer-segment membranes, and its depletion accompanies photoreceptor degeneration in retinal degeneration models [167,168]. However, clinical trials have not demonstrated clear benefits of DHA supplementation, with high-dose DHA failing to significantly slow ERG decline in X-linked RP or improve visual function in Best disease [139,145]. Moreover, the six double bonds of DHA make it highly susceptible to lipid peroxidation, and under strong photo-oxidative conditions, DHA may itself contribute to oxidative injury [169,170]. Therefore, DHA should be considered a nutritional lipid that modulates retinal membrane, metabolic, and inflammatory homeostasis rather than a direct ROS-scavenging antioxidant, and its clinical benefit in IRDs remains uncertain.

Overall, nutritional supplementation is mechanistically plausible, but its clinical efficacy has been inconsistent. A recent American Academy of Ophthalmology assessment concluded that no high-quality evidence currently supports dietary supplementation as an effective disease-modifying treatment for RP, emphasizing the need for adequately powered, genotype-informed trials with clinically meaningful endpoints [171]. In addition, vitamin A is not considered here as a general antioxidant intervention because its role is related to retinoid and visual-cycle metabolism; moreover, excessive supplementation may be undesirable in Stargardt disease because of concerns regarding bisretinoid accumulation [172].

#### 4.1.3. Polyphenols and Other Natural Bioactives

Unlike classical micronutrients, polyphenols and other natural bioactive molecules exert pleiotropic effects that extend beyond direct radical scavenging and include modulation of inflammatory signaling, mitochondrial function, proteostasis, apoptosis, and endogenous antioxidant responses [173]. Several such interventions have progressed from experimental retinal-degeneration models to exploratory human studies, although the strength of clinical evidence varies considerably.

Lycium barbarum (goji berry) has been investigated extensively for its retinal neuroprotective properties, with polysaccharides representing one of its major bioactive fractions. Protective effects of lycium barbarum against ROS accumulation, mitochondrial injury, and photoreceptor loss have been demonstrated in light-induced retinal degeneration models [174]. Subsequently, in a 12-month randomized placebo-controlled trial involving 42 RP patients, oral lycium barbarum supplementation preserved high- and low-contrast visual acuity and slowed macular thinning, but visual-field sensitivity and full-field ERG did not differ from placebo [140]. Although these findings suggest possible neuroprotection of lycium barbarum, the small sample size and use of a complex botanical extract necessitate larger independent trials to confirm efficacy and the contribution of specific constituents to any therapeutic effect.

Chlorogenic acid attenuated hypoxia- and nitric-oxide-associated retinal cell death and suppressed apoptotic signaling in experimental retinal injury models [175,176]. In a prospective single-arm study of 18 RP patients receiving chlorogenic acid 60 mg/day for 3 months, multifocal ERG outer-ring amplitude showed a modest increase, while BCVA, visual-field sensitivity, conventional ERG, and contrast sensitivity remained unchanged [141]. Because of the small sample size, short follow-up, lack of placebo control and no evidence of structural preservation, this observation remains exploratory.

Research on the antioxidant and retinal-protective effects of saffron has faced similar challenges. Fernández-Sánchez et al. and Yamauchi et al. found that the saffron constituents safranal and crocetin preserved photoreceptor morphology and improved ERG responses in *Rho-P23H* rats and protected retinal cells against oxidative and ER stress, respectively [177,178]. In a randomized placebo-controlled crossover trial of 31 patients with Stargardt disease, central focal-ERG amplitude remained stable with saffron during the first 6-month period but declined with placebo [143]. However, this effect was not consistently reproduced after crossover, with no improvement observed in visual acuity and ERG phase [143].

Several other natural bioactives remain at the preclinical stage. Resveratrol reduced oxidative damage and photoreceptor degeneration in *Rpe65*-deficient mice, although its effects involve multiple SIRT1-, Nrf2-, mitochondrial, and inflammatory pathways [135]. Melatonin delayed degeneration in *rd10* mice primarily in association with reduced microglial activation, reactive gliosis, inflammation, and apoptosis [179], whereas curcumin protected *Rho-P23H* photoreceptors mainly by reducing mutant-protein aggregation and ER stress [180]. Because none of these approaches has established clinical efficacy in IRDs, they are not discussed further here.

### 4.2. Endogenous Antioxidant Pathway Activation

#### 4.2.1. Direct Enhancement or Mimicking of Antioxidant Catalytic Activity

Enhancing endogenous ROS-detoxifying enzymes, including SODs, glutathione peroxidases (GPXs), and CAT, represents another antioxidant strategy [181]. These enzymes cooperate to eliminate superoxide radicals, hydrogen peroxide, and lipid peroxides across different cellular compartments, thereby maintaining retinal redox homeostasis [182]. In *rd10* mice, coordinated enhancement of mitochondrial SOD2 and CAT protected cones, further illustrating the importance of compartment-specific and balanced antioxidant activity (also discussed in Section 4.2.4) [183]. However, direct administration of antioxidant enzymes is limited by poor bioavailability and unfavorable pharmacokinetics, leading to the development of enzyme mimetics and catalytically active nanomaterials [181].

Small-molecule pharmacological agents can enhance antioxidant capacity by mimicking the catalytic activity of endogenous antioxidant enzymes. TEMPOL (SOD-mimetic) preserved photoreceptor structure in light-induced degeneration [184]. Ebselen (GPx-mimetic) reduced ROS, enhanced Nrf2 responses, and attenuated glial/vascular injury in retinal models [185,186]. EUK-134 (SOD/CAT-mimetic) suppressed mitochondrial ROS and apoptosis in sodium-iodate-induced RPE injury with structural preservation [187,188]. Together, these studies illustrate the feasibility of pharmacologically reproducing endogenous antioxidant-enzyme activity without delivering the enzymes themselves. However, most evidence comes from light-induced, hypoxic, or chemically induced oxidative-injury models rather than IRDs, and their efficacy, ocular pharmacokinetics, and long-term safety in IRDs remain to be established.

Cerium oxide nanoparticles (CeNPs), one of the most extensively studied nanozymes in ophthalmology, are redox-active nanozymes capable of exhibiting SOD- and CAT-like activities through reversible Ce^3+^/Ce^4+^ cycling [189]. Importantly, their redox behavior is context-dependent, and pro-oxidant effects have also been reported depending on surface chemistry, oxidation state, pH, and dose [190,191]. Appropriately engineered or formulated CeNPs have been shown to reduce oxidative damage, photoreceptor apoptosis, and retinal degeneration in light-induced injury mice and *Rho-P23H* rats [192,193,194]. Despite these protective effects, their long-term persistence in ocular tissues remains an important safety concern, as limited clearance may lead to prolonged tissue exposure [195,196].

#### 4.2.2. Nrf2-Centered Pathway Activation

Nrf2 acts as an upstream coordinator of multiple antioxidant systems rather than as an isolated antioxidant pathway. Upon activation, Nrf2 induces antioxidant response element (ARE)-dependent genes involved in GSH synthesis and recycling, thioredoxin-dependent redox control, peroxide detoxification, and phase II detoxification enzymes such as NQO1 and HMOX1 [197,198,199]. Thus, Nrf2 activation functionally overlaps with the GSH- and thioredoxin-dependent redox systems, which are discussed in Section 4.2.3.

Pharmacological activation of Nrf2 has also shown protective effects in experimental retinal degeneration. In *RHO-P347L* transgenic rabbits, the Nrf2 activator RS9 increased Nrf2-target gene expression and attenuated outer nuclear layer thinning [200,201]. Similarly, dimethyl fumarate (DMF) increased Nrf2 transcriptional activity and reduced mitochondrial ROS in *Rpgr*^−/−^ mice [100]. Sulforaphane has also shown Nrf2-dependent retinal protection in models of RPE oxidative stress [202]. These findings support Nrf2 as an upstream redox-modulating target, although clinical efficacy and long-term safety in IRDs remain unestablished.

In addition to pharmacological activation, AAV-mediated *Nrf2* delivery may enable more sustained and cell-type-selective enhancement of retinal antioxidant defenses. Nrf2 overexpression prolonged cone survival and preserved cone outer-segment opsin localization in *rd1*, *rd10*, and *Rho*^−/−^ mice [203], while RPE-specific expression driven by the Best1 promoter preserved RPE integrity and reduced secondary cone disruption in *rd1* mice [204]. More recently, RPE-targeted AAV-*Nrf2* also protected the RPE, photoreceptors, and retinal vasculature in an oxidative-injury model, partly through growth differentiation factor 15 (GDF15)-mediated paracrine signaling, further illustrating the potential advantage of spatially targeted Nrf2 activation [205].

#### 4.2.3. Thioredoxin and Glutaredoxin Systems

The thioredoxin (TRX) and glutaredoxin (GRX) systems represent downstream redox-effector networks that partly intersect with Nrf2-dependent antioxidant regulation [198]. Nrf2 can transcriptionally reinforce components of the glutathione system [206], whereas direct modulation of TRX or GRX acts more proximally on protein-thiol redox homeostasis [13]. In retinal degeneration, impairment of these systems may exacerbate oxidative damage and accelerate photoreceptor and RPE dysfunction. TRX has been shown to preserve retinal structure and function in *rd1* mice and mice subjected to light-induced retinal injury [207,208]. Similarly, GRX1 overexpression protects RPE cells from oxidative stress-induced apoptosis and alleviates H_2_O_2_-induced oxidative injury [209,210]. Although evidence remains preclinical, these studies suggest that preserving thiol redox homeostasis may be as important as directly scavenging ROS.

#### 4.2.4. Mitochondrial Redox Modulation

Mitochondria are an important source of ROS in metabolically active retinal cells and may provide a spatially defined target for redox-modulating therapy. In *rd10* mice, inducible coexpression of mitochondrial SOD2 and CAT in photoreceptors reduced oxidative damage and preserved cones, whereas either enzyme alone was ineffective [183]. This finding emphasizes the need for coordinated removal of both superoxide and its downstream product H_2_O_2_ rather than indiscriminate suppression of individual ROS. EUK-134 has similarly reduced mitochondrial ROS and apoptosis in experimental RPE oxidative injury [187,188], although its efficacy in IRD models remains to be established. Thus, decreasing mitochondrial ROS can provide retinal protection under selected conditions.

Notably, mitochondrial ROS was not elevated in several models of visual dysfunction without progressive photoreceptor loss [100], indicating that increased mitochondrial ROS is not a universal feature of retinal dysfunction. Therefore, mitochondria-directed therapy should be tailored to genotype, disease mechanism, and stage rather than applied uniformly across IRDs.

#### 4.2.5. Metabolic–Redox Coupling Approaches

Redox regulation in photoreceptors is closely linked to cellular metabolism. This relationship is illustrated by the two proteins encoded by *NXNL1*: RdCVF and RdCVFL. RdCVF promotes glucose uptake and aerobic glycolysis in cones, whereas RdCVFL has TRX-like redox activity [117,118]. AAV-mediated co-delivery of human RdCVF and RdCVFL (AAV-hRdCVF/hRdCVFL) has been proved to preserve cone photoreceptors and delay vision loss in *rd10* mice [211].

TXNIP is a redox-linked metabolic regulator rather than a conventional antioxidant factor. TXNIP binds reduced TRX and participates in the regulation of glucose utilization [120]. Oxidative stress-induced downregulation of TXNIP impairs RPE cell proliferation, disrupts intercellular junctions, and compromises BRB integrity [121].

### 4.3. Limitations of Current Antioxidant and Redox-Modulating Strategies

Despite extensive preclinical evidence, the therapeutic window of antioxidant interventions is often narrow and context-dependent. ROS are not purely harmful as they participate in physiological signaling, and excessive suppression may disrupt redox homeostasis [12]. Moreover, several agents exhibit dose- or environment-dependent effects rather than uniformly antioxidant activity. For example, DHA can promote lipid peroxidation under strong oxidative conditions [169], while CeNPs may act as pro-oxidants depending on specific surface chemistry, oxidation state, concentration, and local microenvironment [190]. Clinical studies further illustrate that increasing antioxidant exposure does not necessarily improve outcomes: in a large, randomized RP trial, high-dose vitamin E was associated with a faster decline in cone ERG amplitude rather than protection [142]. These observations emphasize that antioxidant therapy requires careful dose optimization and should aim to restore, rather than over-suppress, redox balance.

Another challenge is the chronic and progressive nature of IRDs, which may require long-term treatment. Since IRDs arise from genetic defects, antioxidants alone cannot address the primary defect, and disease progression may therefore continue despite partial mitigation of oxidative damage [2,12]. Orally administered antioxidants face limited retinal bioavailability, adherence issues, and cumulative safety concerns [137]. In addition, oxidative stress sources vary with genotype and disease stage, making a fixed antioxidant plan unlikely to be uniformly effective. These issues may partly explain inconsistent clinical translation. DHA supplementation failed to significantly slow overall disease progression in RP trials [139] and showed no significant functional benefit in Best disease [145]. Similarly, lutein did not meet the primary visual-field endpoint in RP patients, although a secondary mid-peripheral visual-field endpoint suggested a modest benefit [138]. Thus, apparently positive subgroup or secondary-endpoint findings should be interpreted cautiously until reproduced in prospectively powered trials.

Finally, the clinical evidence supporting many antioxidant or redox-modulating approaches remains limited by study design. Several human studies have involved small cohorts, short follow-up, surrogate functional endpoints, or uncontrolled designs. For example, chlorogenic acid (18 patients, short-term, single-arm study) showed only selected multifocal ERG parameters and no significant change in BCVA, conventional ERG, or visual field [141]. The lycium barbarum study (42 patients) showed signals in selected visual and structural measures but not in visual-field or full-field ERG outcomes [140], while the saffron trial in Stargardt disease (31 patients) provided only limited short-term functional evidence [143]. Future trials should therefore adopt adequately powered and controlled designs, incorporate stage-specific patient selection, prespecified clinically meaningful endpoints, and include long-term follow-up for durability and safety.

## 5. How Antioxidant Strategies Could Be Integrated with Gene Therapy

Given the complementary mechanisms of gene therapy and antioxidant therapy, the next step is to explore whether combining them could yield better and longer-lasting outcomes.

### 5.1. Phase-Specific Integration of Antioxidant Strategies with Gene Therapy

Antioxidant therapy can be strategically integrated at different phases of the gene therapy timeline. Before gene therapy, antioxidant treatment may help preserve viable photoreceptors and RPE cells and thereby extend the therapeutic window for genetic rescue. The efficacy of many gene-directed therapies depends on the presence of sufficient surviving target cells, making advanced retinal degeneration an important constraint on treatment response [56]. By reducing ongoing oxidative injury and slowing secondary cellular degeneration, antioxidant or redox-modulating interventions may help maintain retinal integrity before gene delivery and potentially preserve a larger population of cells capable of responding to subsequent genetic correction. Such pretreatment may therefore be particularly relevant in slowly progressive IRDs or in patients approaching the lower limit of viable target-cell preservation, although the extent to which antioxidant pretreatment can expand eligibility for gene therapy remains to be established clinically.

Concurrent administration provides an opportunity to combine genetic rescue with immediate local redox protection. A recent preclinical study provided direct proof of concept for this strategy in *rd10* mice, in which an AAV vector carrying the therapeutic *Pde6b* gene was covalently conjugated to the ROS-scavenging catalytic G-quadruplex–hemin DNAzyme CoG4 [15]. The combined AAV–CoG4 platform produced greater structural and functional preservation than either component alone, supporting the potential benefit of synchronizing restoration of the primary genetic defect with local control of oxidative stress [15]. Notably, CoG4 alone delayed outer nuclear layer degeneration but did not restore retinal function, suggesting that redox modulation is more likely to complement than replace gene-directed rescue. This approach also illustrates a broader principle: concurrent antioxidant support may be most useful when oxidative injury persists during the period in which genetic rescue is being established [15].

After gene therapy, continued redox modulation may help preserve retinal stability and sustain therapeutic benefit, particularly when the primary genetic intervention does not fully arrest downstream degeneration. Even after successful gene delivery, pre-existing cellular stress, retinal remodeling, metabolic imbalance, or inflammatory responses may continue to influence disease progression [56]. Antioxidant treatment could therefore serve as a maintenance strategy by limiting secondary oxidative injury and supporting cellular homeostasis during post-treatment adaptation. However, the contribution of treatment-associated factors such as transgene-induced ER stress, vector-associated inflammation, or delivery-related mechanical injury to oxidative stress in the treated retina has not been fully established. Post-treatment antioxidant therapy should therefore be viewed as a mechanistically plausible adjunct and requires further experimental and clinical validation.

### 5.2. Disease-Specific Integration Strategies

Because oxidative stress arises from different upstream disturbances across IRDs, its integration with gene therapy is unlikely to follow a uniform model. The optimal combination strategy should therefore depend on the causative gene, the cellular source and stage of oxidative stress, the target cell of gene therapy, and whether redox dysregulation acts as a primary driver or a secondary amplifier of degeneration.

In RP patients, antioxidant therapy may be particularly useful as an adjunct to gene therapy after substantial rod loss, when reduced oxygen consumption produces relative hyperoxia and oxidative stress in surviving cones [126]. Gene-directed therapy addresses the primary molecular defect but requires sufficient preservation of target cells, whereas redox modulation may help maintain cone viability and thereby extend the therapeutic window [128]. The clinical development of NAC in RP provides a translational basis for this concept, although whether antioxidant treatment improves the efficacy or durability of gene therapy has not yet been tested directly.

In Stargardt disease, oxidative stress arises more directly from abnormal retinoid handling and accumulation of photoreactive bisretinoids [130]. Consequently, combination strategies should preferentially target this upstream source of oxidative injury rather than rely solely on nonspecific ROS scavenging. Gene correction or replacement could restore ABCA4 function, whereas adjunctive approaches that reduce vitamin A dimerization, bisretinoid formation, or photooxidative stress may limit ongoing RPE injury before and after genetic rescue.

LCA further illustrates that combination strategies must be genotype-specific even within the same clinical diagnosis. In *Rpe65* deficiency, oxidative stress appears to act mainly as a secondary amplifier [134]. Therefore, gene augmentation remains the central therapeutic intervention, with antioxidant treatment serving, at most, as a complementary approach to preserve stressed photoreceptors. In contrast, *NMNAT1*-associated degeneration is accompanied by prominent oxidative DNA damage, and N-acetylcysteine reduced DNA damage and preserved photoreceptors in *Nmnat1*-mutant mice [16]. Redox modulation may therefore represent a more proximal therapeutic target in *NMNAT1*-associated disease than in *RPE65* deficiency, although clinical evidence is currently lacking.

Taken together, antioxidant and redox-modulating therapies may be integrated with gene therapy at different stages for distinct purposes: preserving target cells before treatment, providing complementary protection during genetic rescue, and limiting secondary degeneration after treatment. Rather than representing a uniform adjunct applied throughout the treatment course, their optimal use is likely to depend on disease mechanism, stage of degeneration, source of oxidative stress, and the specific gene therapy platform. Combining antioxidant and gene therapy may become a more reasonable and practical strategy, providing a new direction for achieving more stable and durable treatment effects in patients with IRDs.

### 5.3. Translational Considerations Before Combining Antioxidant and Gene Therapy for IRDs

Despite the promising prospects of combining antioxidant therapy with gene therapy, the lack of reliable biomarkers, complexity of clinical trial design, selection of optimal antioxidant agents, and concerns over viral vector safety and efficacy all pose challenges to the clinical practice of combining antioxidant therapy with gene therapy. In addition, heterogeneity among patients with IRDs makes it difficult to achieve ideal results only through a single treatment model. The following sections discuss each of these key challenges in turn, focusing on moving antioxidant–gene therapy combinations toward clinical application.

#### 5.3.1. Biomarker Development

The lack of validated, clinically feasible biomarkers to accurately monitor retinal redox status remains a major obstacle to the clinical translation of antioxidant therapies. Unlike systemic oxidative stress indicators, retinal oxidative damage is highly localized and dynamically regulated [12], making it difficult to quantify accurately by conventional blood-based assays. At present, exploratory approaches reported in IRDs and related optic neurodegenerative disorders include measurements of oxidative metabolites in ocular fluids, metabolic or autofluorescence-based imaging readouts, and molecular signatures derived from transcriptomic studies [212,213,214,215]. However, most of these approaches have not been validated as quantitative biomarkers of retinal redox status in IRDs.

#### 5.3.2. Clinical Trial Design and Endpoint Selection

The design of clinical trials for combination therapy in IRDs is naturally challenging due to the complex natural course of the disease, significant genotypic and phenotypic heterogeneity, and large differences in disease progression rates among different patients [216]. In particular, structural changes and functional outcomes often do not change in parallel. For example, in multiple gene therapy studies, patients may show long-term improvement in visual function despite continued retinal degeneration [59]. This structure-function inconsistency complicates the selection of treatment endpoints and may also mask the additional benefit of adjuvant antioxidant therapy.

Future clinical trials may need to use multimodal endpoints that integrate visual-function assessments, high-resolution imaging measures, and molecular biomarkers to more fully capture the true effect of combination therapy.

#### 5.3.3. Choosing the Appropriate Antioxidant Adjunct

Not all antioxidant strategies are biologically equivalent; thus, selecting the most appropriate adjuvant modality remains a critical challenge. The therapeutic effect of antioxidant intervention depends not only on the drug or molecular type used, but also on the specific type, concentration, subcellular localization of ROS, as well as the timing and dose of treatment [181].

Direct ROS scavengers may be particularly suitable for reducing acute oxidative load. However, excessive inhibition of ROS may interfere with physiological redox signaling essential for cellular homeostasis [217]. Similarly, enzymatic antioxidant therapy also requires careful consideration of the balance between different antioxidant pathways. For example, SOD can convert O_2_^•−^ to H_2_O_2_; without adequate downstream scavenging by CAT or GPXs, the accumulation of H_2_O_2_ may further aggravate oxidative damage [218].

#### 5.3.4. Vector Engineering, Safety, and Long-Term Durability

Antioxidant–gene therapy combinations face major technical and safety challenges. Dual or multifunctional vector strategies must balance payload capacity, transduction efficiency, and cell-type specificity, while minimizing immunogenicity and off-target effects. Additionally, long-term regulation of antioxidant gene expression is critical, as sustained activation may disrupt physiological redox signaling and induce unexpected toxicity.

Therefore, ensuring vector stability, production scalability, and sustained therapeutic efficacy will be key to driving the clinical translation of this combination strategy.

#### 5.3.5. Toward Personalized Combination Therapy

Ultimately, successfully applying combination therapies in IRDs will rely on a precision medicine framework—tailoring treatment plans to patient genotype, residual retinal structure, disease stage, and oxidative stress load. Achieving this requires refining biomarker systems, compiling comprehensive natural history data, and elucidating the interplay between oxidative stress and disease-specific degenerative pathways.

## 6. Conclusions

Gene therapy has fundamentally changed the treatment paradigm for IRDs by targeting the underlying genetic defects. However, genetic rescue does not necessarily normalize the downstream retinal microenvironment, and oxidative stress may continue to contribute to degeneration in a disease-, genotype-, and stage-dependent manner. Antioxidant and redox-modulating therapies should therefore be viewed as potential complements rather than alternatives to gene-directed treatment. Current evidence for such combinations remains predominantly preclinical, and the contribution of inflammation associated with gene therapy, transgene expression, and delivery-related injury to retinal oxidative stress has not been fully established. Future studies should determine which IRDs and disease stages are most likely to benefit from redox modulation, with particular attention to mitochondrial ROS, retinal redox biomarkers, regulated transgene expression, treatment timing and dose, and long-term safety. Ultimately, a mechanism-guided combination of genetic correction and microenvironmental stabilization may provide a rational route toward more durable therapeutic outcomes in selected patients with IRDs.

## Figures and Tables

**Figure 1:**
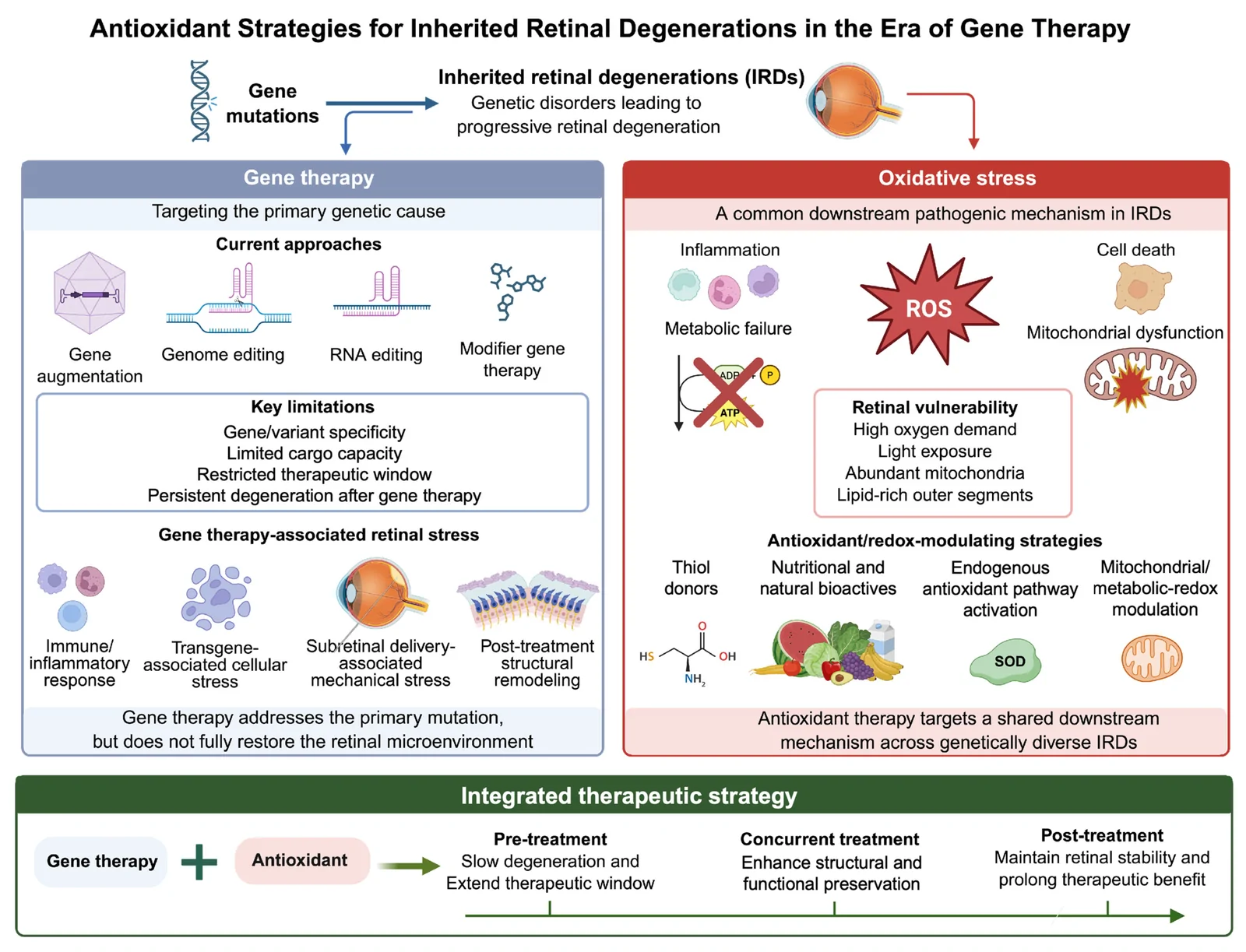
Conceptual framework for integrating antioxidant and redox-modulating strategies with gene therapy in inherited retinal degenerations (IRDs). Gene therapy primarily addresses the underlying genetic defect but is limited by gene/variant specificity, vector capacity, viable target-cell availability, and persistent retinal degeneration, and may also introduce additional retinal stress through immune/inflammatory responses, transgene-associated cellular stress, and subretinal delivery. Oxidative stress represents a shared downstream mechanism that interacts with inflammation, metabolic and mitochondrial dysfunction, and cell-death pathways. Antioxidant and redox-modulating strategies may therefore complement gene therapy at different stages, including pretreatment, concurrent treatment, and post-treatment maintenance. Created in BioRender. Lu, F. (2026) https://BioRender.com/onxws6h.

**Table 1:** Classification and mechanisms of gene therapy strategies for inherited retinal degenerations (IRDs).

	Classification	Mechanism	Genome Modification	Causative Gene/Variant Specificity	Representative Disease/Gene Targets
DNA-targeted	Gene augmentation	Delivery of a functional gene copy to restore protein expression	No	Gene-specific variant-independent	*RPE65*-associated LCA2 [20]; *RS1*-associated X-linked retinoschisis [21]; *RLBP1*-associated retinal dystrophy [22]; *PRPF31*-associated RP [23]
	Genome editing	Permanent modification of pathogenic DNA variants using programmable nucleases, including CRISPR/Cas9, base editors, and prime editors	Yes	Variant-specific	*CEP290*-associated LCA10 [24]; *USH2A*-associated IRD [25,26]; Stargardt disease [27]; *RHO*-associated autosomal dominant RP [28]
RNA-targeted	RNA editing/RNA-targeted correction	Sequence-specific correction or modulation of mutant transcripts using programmable RNA-targeting systems	No	Variant-specific	*USH2A*-associated IRD [29]; Stargardt disease [30]
	Antisense oligonucleotide therapy	Modulation of RNA processing through sequence-specific oligonucleotides, primarily by correcting aberrant splicing, altering transcript stability, or regulating translation	No	Variant-specific	*CEP290*-associated LCA10 [31]; *USH2A*-associated IRD [32]; *RHO*-associated autosomal dominant RP [33]
	Translational readthrough therapy	Promotion of ribosomal bypass of premature termination codons to restore production of full-length functional proteins	No	Variant-specific (nonsense variants)	*RPE65*-associated LCA2 [34]; *KCNJ13*-associated LCA16 [35]
Pathway-modulating	Modifier-gene therapy	Introduction of regulatory genes or protective factors that modulate disease-associated pathways downstream or independent of the causative gene	No	Causative gene/variant-independent	Multiple models of retinitis pigmentosa [36]
Cell function-restoring	Optogenetic therapy	Introduction of light-sensitive proteins into surviving retinal cells to restore light responsiveness independent of photoreceptor function	No	Causative gene/variant-independent	Advanced retinitis pigmentosa; late-stage IRDs [37,38]

**Table 2:** Clinical evaluation of antioxidant and redox-modulating interventions in IRDs.

Disease	Intervention	Mechanism	Human Evidence	Clinical Trial Status	References
RP	N-acetylcysteine (NAC)	A cysteine and glutathione (GSH) precursor. Increases intracellular GSH and reduces reactive oxygen species (ROS).	30 patients with RP, oral NAC, 6 months: Generally well tolerated, with modest signals of improvement in best-corrected visual acuity (BCVA) and macular sensitivity.	Phase I (NCT03063021) completed; Phase III (NCT05537220) active, not recruiting.	[17,137] NCT03063021 NCT05537220
RP	Lutein	A macular pigment carotenoid with antioxidant activity. Filters short-wavelength blue light, quenches singlet oxygen, and may reduce lipid peroxidation and photo-oxidative stress.	225 nonsmoking patients with RP, lutein 12 mg/day plus vitamin A, 4 years: The primary endpoint was negative, whereas the secondary endpoint (HFA 60-4 mid-peripheral visual field) suggested a modestly slower rate of decline.	Phase III (NCT00346333) completed.	[138]
X-linked RP	Docosahexaenoic acid (DHA)	A major lipid constituent of photoreceptor outer-segment membranes. May modulate membrane homeostasis, inflammation, and lipid peroxidation; Highly susceptible to lipid peroxidation, may contribute to oxidative injury under strong photo-oxidative conditions.	78 patients with X-linked RP, DHA 30 mg/kg/d plus multivitamin, compared with placebo: no difference in the rate of cone electroretinography (ERG) functional loss, no evident difference for change in rod ERG, maximal ERG, or cone implicit time.	Phase II (NCT00100230) completed.	[139]
RP	Lycium barbarum (goji berry) supplement/polysaccharides	May have protective effects against ROS accumulation, mitochondrial injury, and photoreceptor loss.	42 patients with RP, 2 packs/day (each containing 5 g net weight), 12 months: Some visual and macular structural measures suggested stabilization or protection, no significant improvement in visual-field and full-field ERG outcomes.	Clinical trial (NCT02244996) completed.	[140]
RP	Chlorogenic acid	A polyphenolic antioxidant. May reduce ROS generation, lipid peroxidation, and inflammatory signaling.	18 patients with RP, 60 mg/day, 3 months: A modest increase in multifocal ERG outer-ring amplitude, while no improvement in BCVA, visual-field sensitivity, conventional ERG, and contrast sensitivity.	Human interventional study (not a randomized controlled trial), completed.	[141]
RP	Vitamin E	A lipid-soluble chain-breaking antioxidant.	601 patients with RP, 4 groups, 4–6 years: The two groups receiving 400 IU/d of vitamin E were more likely to have a decline in cone ERG amplitude.	Randomized, controlled, double-masked trial with 2 × 2 factorial design, completed.	[142]
Stargardt disease	Saffron (crocin/crocetin)	Saffron-derived carotenoids with antioxidant, anti-inflammatory, and anti-apoptotic properties. May counter photo-oxidative damage associated with ABCA4 dysfunction.	31 patients with Stargardt disease, saffron 20 mg/day, 6 months: Focal ERG showed functional signals, long-term slowing of retinal atrophy not demonstrated.	Phase I/II (NCT01278277), status on ClinicalTrials listed as unknown, results published.	[143]
Stargardt disease/*ABCA4*-associated cone-rod dystrophy	Lutein	Increases macular pigment, filters short-wavelength light, and quenches ROS. Theoretically may reduce photo-oxidation of A2E/A2-PE.	17 patients with Stargardt disease or cone-rod dystrophy, lutein 20 mg/day, 6 months: Serum lutein and macular pigment optical density increased in some participants, no improvement in central visual function.	Open-label human pilot study (not a randomized controlled trial), completed.	[144]
Best disease	DHA	A major lipid constituent of photoreceptor outer-segment membranes. May modulate membrane homeostasis, inflammation, and lipid peroxidation; Highly susceptible to lipid peroxidation, may contribute to oxidative injury under strong photo-oxidative conditions	8 patients with Best disease, DHA 20 mg/kg/day: Plasma DHA increased two- to threefold, no significant improvements in visual acuity, VF-14, or the electrooculography Arden ratio.	Small randomized, double-masked, placebo-controlled crossover clinical trial, completed.	[145]
Usher syndrome	N-acetylcysteine amide (NACA; NPI-001)	A cell-permeable cysteine derivative. Increases intracellular cysteine and GSH levels; Stimulates the expression and activity of nuclear factor erythroid 2-related factor 2 (Nrf2)	49 participants, oral NPI-001, 24 months: (Sponsor-reported) > 50% reduction in photoreceptor loss measured by EZ area, with generally favorable tolerability	Phase I/II (NCT04355689) completed; Phase III (NCT07290530 and NCT07710196) not yet recruiting *.	Sponsor website (https://www.nacuity.com) NCT04355689 NCT07290530 NCT07710196
Usher syndrome	Lutein	Increases macular pigment, filters short-wavelength light, and quenches ROS.	11 patients with Usher syndrome, lutein 20 mg/day, 6 months: Increased serum lutein and macular pigment density in some eyes, no improvement in central visual function.	Human supplementation study (not a formal randomized placebo-controlled efficacy trial), completed.	[146]

* Clinical-trial status was verified against ClinicalTrials.gov on 14 September 2026.

## Data Availability

No new data were created or analyzed in this study. Data sharing is not applicable to this article.

## Abbreviations

The following abbreviations are used in this manuscript:

AAV	Adeno-associated virus
ARE	Antioxidant response element
BCVA	Best-corrected visual acuity
BRB	Blood–retinal barrier
CAT	Catalase
CeNPs	Cerium oxide nanoparticles
CoG4	Catalytic G-quadruplex-hemin DNAzyme
CRISPR	Clustered regularly interspaced short palindromic repeats
DHA	Docosahexaenoic acid
DMF	Dimethyl fumarate
ER	Endoplasmic reticulum
ERG	Electroretinography
EZ	Ellipsoid zone
FDA	Food and Drug Administration
GDF15	Growth differentiation factor 15
GSH	Glutathione
GPXs	Glutathione peroxidases
GRX	Glutaredoxin
IRD/IRDs	Inherited retinal degeneration(s)
LCA	Leber congenital amaurosis
NAC	N-acetylcysteine
NACA	N-acetylcysteine amide
NACET	N-acetyl-L-cysteine ethyl ester
Nrf2	Nuclear factor erythroid 2-related factor 2
NOX	NADPH oxidase
RdCVF	Rod-derived cone viability factor
RdCVFL	Rod-derived cone viability factor long
RPE	Retinal pigment epithelium
ROS	Reactive oxygen species
RP	Retinitis pigmentosa
SOD	Superoxide dismutase
TRX	Thioredoxin
TXNIP	Thioredoxin-interacting protein
